# Exploring factors affecting psychological resilience of farmers living in drought-affected regions in Iran: a qualitative study

**DOI:** 10.3389/fpsyg.2024.1418361

**Published:** 2024-08-27

**Authors:** Azadeh Tahernejad, Sanaz Sohrabizadeh, Ali Mashhadi

**Affiliations:** ^1^Department of Health in Emergencies and Disasters, School of Public Health and Safety, Shahid Beheshti University of Medical Sciences, Tehran, Iran; ^2^Air Quality and Climate Change Center, Research Institute for Health Sciences and Environment, Shahid Beheshti University of Medical Sciences, Tehran, Iran; ^3^Faculty of Education and Psychology, Ferdowsi University of Mashhad, Mashhad, Iran

**Keywords:** psychological resilience, drought, farmers, Iran, qualitative study

## Abstract

**Introduction:**

Drought, a prevalent consequence of climate change, significantly impacts mental health among farmers. Enhancing psychological resilience is crucial to mitigating these effects. This study aims to explore the concept and factors affecting the psychological resilience of farmers living in drought-affected regions in Iran.

**Method:**

This study utilized a conventional qualitative content analysis method. Twenty-six participants, divided into two groups of experts and farmers, were selected through purposive sampling. Data collection was conducted via in-depth semi-structured interviews. The study adhered to the Consolidated Criteria for Reporting Qualitative Research (COREQ) checklist and was carried out between June 2023 and February 2024.

**Results:**

The factors affecting the psychological resilience of farmers living in drought-affected regions were categorized into two main categories with nine subcategories. The first category, environmental factors, included economic factors, socio-cultural factors, drought adaptation methods, government policies, and infrastructural factors. The second category, intra-individual factors, comprised personal characteristics, health factors, psychological factors, and perception and knowledge.

**Conclusion:**

The study revealed that both environmental and intra-individual factors influence the psychological resilience of farmers during droughts. It is recommended to implement intervention strategies, such as providing training and education on drought adaptation methods and managing agricultural and healthcare expenses. Further research is needed to expand this concept to various disasters and occupational groups in future studies.

## 1 Introduction

Climate Change (CC) (2013) includes any alterations in climate patterns over time, whether stemming from natural variability or human activities. Nowadays, the global community faces a growing number of severe incidents and calamities triggered by climate change (Thomas, 2017). These changes have significant repercussions, such as increasing the occurrence and intensity of drought in numerous regions worldwide. A common and prevalent disaster caused by climate change consequences is drought (Mukherjee et al., 2018). Furthermore, climate change can potentially modify precipitation patterns, resulting in more pronounced dry periods in specific regions and intense rainfall in others. This shift can lead to water shortages for human, agricultural, and industrial purposes, bringing about substantial social and economic consequences (Pereira et al., 2021).

Droughts are categorized as gradual disasters that impact human health and well-being, causing significant damage to agricultural, hydrological, and environmental systems (Mishra and Singh, 2010). Drought has roots in antiquity and has been a persistent challenge throughout human history. In the contemporary era, global warming and climate change, exacerbated by unsustainable human activities, have emerged as paramount concerns, aggravating the existing causes of drought and other natural disasters like floods and storms. Unlike earthquakes, drought unfolds gradually over an extended period, making it challenging to pinpoint its onset, duration, and geographical scope accurately (Ault, 2020). The direct consequences of drought are primarily linked to climatic conditions, weather patterns, and ecological characteristics. Conversely, the indirect impacts of drought, which are broader and more nuanced, encompass economic, environmental, social, and health-related damages that are complex to quantify. For instance, economic impacts include income reduction with cascading effects. Environmental consequences encompass the degradation of plant and animal species, diminished water and air quality, and soil erosion (Tramblay et al., 2020). The social impacts of drought extend to health implications, decreased quality of life, and compromised public safety. Many factors recognized as economic and environmental implications also have health and social dimensions (Edwards et al., 2019).

One of the most critical consequences of drought is the escalation of psychological strains (Manning and Clayton, 2018). There is strong theoretical basis to anticipate that drought can have adverse effects on mental health. Among various impacts, we can highlight the economic implications resulting in difficulty and stress, the breakdown of social connections due to the harmful effects of drought, and the distress caused by witnessing harm to livestock, crops, soil, and indigenous vegetation (Lindvall et al., 2020).

The agricultural sector is often affected by drought because of its significant dependence on water resources and soil moisture retention (Jiménez-Donaire et al., 2020). Drought is a significant meteorological occurrence posing a substantial threat to agriculture (Sun et al., 2019). Farmers constitute a crucial group, that is significantly impacted by the consequences of diminished mental health during drought periods. Published reports provided evidence indicating that the severity of drought correlates with a more severe impact on farmers’ mental health (Edwards et al., 2015). The psychological disorders due to drought on farmers can be profound (Savari et al., 2021). One study revealed that farmers experiencing complete or partial loss of farm productivity due to drought were 8.49 times more likely to report mental health issues than those unaffected by drought (Edwards et al., 2015).

Another research study revealed that in Australia, a male farmer dies by suicide approximately every 4 days. This rate of suicide is notably higher than the general population, raising significant public health concerns (Page and Fragar, 2002). Elevated suicide rates have also been documented in agricultural communities in the United Kingdom (Hounsome et al., 2012), France (Bossard et al., 2016), Japan (Tsutsumi et al., 2007), Canada (Jones-Bitton et al., 2020), and the United States (Reed and Claunch, 2020). This concerning pattern is not limited to developed nations like Australia; it is also evident in developing countries such as India, where there is a link between crop failures resulting from droughts and suicide attempts among farmers (Merriott, 2016; Mishra, 2012).

Iran is situated within arid and semi-arid climates globally (Alvankar and Fattahi, 2016). By 2039, a significant portion of the country is projected to confront severe to extremely severe drought conditions (Khazanedari et al., 2009). Small-scale agriculture plays a pivotal role in Iran (Fotohabadi and Zamani, 2018), with agriculture being the most significant economic sector following the service industry, contributing approximately 26% to the gross domestic product (Zand and Mosavi, 2022). With over 90% of Iran’s regions experiencing varying degrees of drought (Abdollahi et al., 2022), nearly all Iranian farmers are contending with the effects of drought to some extent. The recurrent droughts in arid and semi-arid rural areas not only pose numerous challenges for farmers but also impact their mental health. Research findings indicate that many Iranian farmers and their families have either left the agricultural sector or lost their vitality and liveliness due to the recent droughts (Savari and Khosravipour, 2018). Additionally, in a separate study, rural farmers in Yazd province of Iran were found to have low average scores for job and life satisfaction (Jamaati Ardakani, 2017). Various research studies have highlighted adverse effects on farmers’ quality of life, heightened mental stress and disorders, social and familial breakdown, diminished social vigor, feelings of hopelessness, and an aging population among Iranian farmers (Bayad, 2016; Bathaiy et al., 2021; Zarif Moradian et al., 2022; Savari et al., 2023).

Enhancing psychological resilience is a critical approach to mitigating mental health repercussions (Riehm et al., 2021), particularly essential in strengthening farmers’ resilience against drought (Javadinejad et al., 2021). Psychological resilience, a component of overall resilience, encompasses the capacity to withstand crises mentally or emotionally and rapidly restore equilibrium to pre-crisis levels (De Terte and Stephens, 2014). Despite the conducted research, comprehensive insights into the psychological resilience of farmers amidst drought conditions remain scarce. Recognizing the significance of determining the factors affecting farmers’ psychological resilience is crucial for shaping policy initiatives and crafting suitable strategies to alleviate the impacts of declining mental health.

Accordingly, conducting a study to enhance comprehension of the concept and factors influencing farmers’ psychological resilience is essential to answer the question what are the factors affecting the psychological resilience of farmers affected by drought? Hence, this study aimed to explore the factors affecting the psychological resilience of farmers living in drought-affected regions of Iran.

## 2 Materials and methods

### 2.1 Study design

The present study is qualitative research using conventional content analysis (Graneheim and Lundman, 2004) to derive codes, subcategories, and categories. This research aligns with the standards outlined in the Consolidated Criteria for Reporting Qualitative Research (COREQ) checklist and was carried out from June 2023 to February 2024.

### 2.2 Setting

This study was conducted in Iran, situated in the arid and semi-arid Middle East region (Emadodin et al., 2019). Iran consistently faces high levels of drought, albeit with varying features and impacts across different regions (Ahmadyoosefi et al., 2021). Roughly 97% of the country experiences drought (Khazanedari et al., 2009; Doostan, 2020), potentially placing Iran in a challenging position due to significant fluctuations in rainfall and limited water resources (Shahi, 2019). Research results show that drought varies in extent and severity across different regions of Iran, from the north to the south and from the east to the west (Fasihi et al., 2024; Karimi and Heidari, 2023; Nazaripour et al., 2023). To maximize variation, cities were selected from a variety of regions, including Babolsar in the north, Rafsanjan, Bandar Abbas, and Shiraz in the south, Tehran, Karaj, and Firouzkoh in the central region, Qochan and Mashhad in the east, and Ilam in the west. The other reasons for selecting these cities are the widespread and severe drought conditions experienced across almost all parts of the cities and the diverse, multi-product nature of its agricultural sector. These cities include various agricultural and garden lands and various climatic conditions, including hot and dry, temperate, and mountainous, in their regions and villages. Additionally, these cities feature both irrigated and rainfed agricultural systems (Sadeghloo et al., 2020).

### 2.3 Participants

The study participants were chosen using the purposive sampling method, based on which, 26 participants, including experts and farmers. Because interviewing related experts can provide a broader perspective on the research topic. Experts may have a different vantage point or deeper understanding of the context, which can complement the insights from the farmers. Inclusion criteria for experts were considered as having at least a bachelor’s degree, having at least 1 year of research or educational experience in the fields of psychological resilience, drought management, incident and disaster management, disaster psychology, climate change, and drought, sociology of disasters, health policy, and other related fields. Also, inclusion criteria for farmers were male and female farmers occupied in small-scale farming, aged between 18 and 70 years, with a minimum of 3 years of agriculture experience, residing in villages and regions impacted by drought, and actively engaged in farming activities. The exclusion criteria were a lack of interest in further participation.

The number of participants in this research was determined by the data saturation principle. Data saturation was achieved after 24 interviews, and two more interviews were conducted to ensure no new concept would emerge.

### 2.4 Data collection

Data collection was conducted using in-depth semi-structured interviews. Initially, four unstructured interviews were carried out to extract general concepts. Subsequently, utilizing an interview guide, 22 semi-structured face-to-face interviews were conducted. The interviews took place in a quiet setting and comfortable atmosphere to ensure the participant was calm. Every interview started with some open questions followed by more detailed questions to probe the interview. An example of an interview question, to farmers included “From your perspective, what are the consequences of drought that you have encountered?” or “Based on your experiences, what factors contribute to your mental strength in facing the stress and challenges of drought?” Similarly, experts were asked questions such as “Based on your knowledge, what factors impact the psychological resilience of farmers affected by drought?”

A well-trained member of our research team (AT) who had a good ability for data gathering in qualitative research, was responsible for conducting the interviews. All interviews were scheduled in advance, and the interview location was chosen based on the participants’ preferences. The interviews were recorded, after the participants’ consent, and to enhance data accuracy, each interview was transcribed immediately. All interviews were conducted in Persian, the participants’ native language, and lasted from 25 to 45 min, with an average duration of 35 min.

### 2.5 Data analysis

Data analysis was conducted concurrently with data collection, using the content analysis method following the five-step approach of Graneheim and Lundman (2004). Initially, each recorded interview was transcribed verbatim using MS Word software. The transcript was then meticulously reviewed several times to identify sentences reflecting the participants’ experiences, marked as meaning units within the text. These meaning units were condensed and abstracted into codes in the next step. The codes were subsequently organized into subcategories based on their relationships, similarities, and differences. Ultimately, the main categories were formed by classifying these subcategories. This five-step iterative process was applied to all interviews until the main categories were finalized. The first author performed the first three steps. The final two steps of the analysis process involved all authors. Participants did not review the initial content of the interviews or the analysis; however, the researcher took their notes and feedback into account after each interview. Manual methods were used for data handling, with no software employed for analysis. An example of analysis for conceptualization is shown in Table 1. When discrepancies arose between two researchers regarding codes or categories, we consulted the other authors for assistance in data analysis. Reliability was assessed using the formula proposed by Cowan et al. (1997):

$$\mathrm{Reliability}=\mathrm{Number of agreements}/\left(\begin{array}{l} \mathrm{Number of agreements}+\\ \mathrm{Number of disagreements} \end{array}\right)\text{.}$$

**Table 1:** An example of analysis for conceptualization.

Part of the interview	Meaning unit	Code	Subcategory	Category
“Many of the trees in my farm have dried up, and I have not had a harvest even close to what I had a quarter of a year ago…”	Impact of drought on harvest	Reduction of harvest	Economic factors	Environmental Factors
“When I have enough income from my crops, I feel more secure about the future. I can plan and invest in my farm.”	Feeling stable and confident due to sufficient income.	Financial security		
“The lack of proper insurance coverage makes me anxious. Last year, I lost a lot, and the insurance did not help much.”	Anxiety stemming from inadequate insurance coverage and inconsistent reimbursements.	Insurance coverage		
“With rising costs for seeds and fertilizers, I worry about making ends meet. It’s hard to cope when the prices of my products are so low.”	Stress and anxiety are caused by high agricultural expenses and low product prices.	Cost pressure		
“My friends and family always listen to my problems. Particularly my wife truly comprehends me. Their support helps me cope with the stress of farming.”	The role of emotional support from family and friends in managing stress.	Emotional support	Socio-cultural factors	
“Participating in village festivals and traditional ceremonies reminds me of my roots and gives me hope during tough times.”	The importance of cultural traditions in providing purpose and enhancing psychological resilience.	Cultural traditions		
“Being part of local community groups gives me a sense of belonging. We share our challenges and celebrate our successes together.”	Sense of belonging and shared experiences that enhance resilience through community engagement.	Community bonding		

Typically, a minimum agreement percentage of 75% is considered sufficient (Cook, 2012). In this study, the overall agreement among themes, categories, and subcategories was 93%, indicating a strong consensus among the authors. Direct quotes from the interviews were included in the results section to elucidate the codes, subcategories, and categories.

### 2.6 Trustworthiness

The trustworthiness of our study was evaluated using the four strategies such as credibility, dependability, confirmability, and transferability (Korstjens and Moser, 2018). To ensure the credibility of the data, techniques such as prolonged engagement, triangulation, member checks, peer debriefing, and negative case analysis were employed (Anney, 2014). Specifically, the researcher spent 5 months actively involved in the drought field, continuously making observations and compiling field notes. For triangulation, multiple researchers evaluated the study, providing diverse perspectives that enhanced the integrity of the findings. Data triangulation involved using various sources and methods, including interviews and field observations, to improve the quality of the data collected. To conduct member checks, the analyzed and interpreted data were returned to participants for their evaluation, allowing them to suggest changes if they were dissatisfied with the interpretations. Additionally, peer debriefing offered researchers the chance to test their insights and address challenging questions. Negative case analysis was applied when collected data contradicted the researcher’s expectations, ultimately enhancing the study’s credibility. Data dependability was assessed through an audit trail, stepwise replication, code-recode strategies, and peer review. Furthermore, a reflexive journal was maintained, along with documentation of the study, to evaluate the confirmability of the data, taking into account the researcher’s background and interest in the topic (Anney, 2014). Confirmability was achieved by maintaining a neutral stance towards the data and documenting researchers’ opinions on the findings. The current manuscript ensures dependability by providing comprehensive details for other researchers to replicate and extend the study. Data transferability was ensured by offering a comprehensive description of all study stages, including study design, setting, participant selection, data collection, and data analysis (Anney, 2014). By employing maximum variation sampling, the researchers gathered a diverse range of comments, observations, and interpretations.

### 2.7 Ethical consideration

The study was approved by the Ethics Committee of the School of Public Health and Safety at Shahid Beheshti University of Medical Sciences (ethical code: IR.SBMU.PHNS.REC.1402.034). The study objectives were clarified to the participants before conducting the study, and they could withdraw at any stage. Participants signed an informed written consent form regarding the recording of interviews. They were guaranteed confidentiality of information and the anonymity of the participants and their official posts or responsibilities.

## 3 Results

The study included 88% male participants, with the most common educational level being middle school (*n* = 8). The most common age group among interviewees was 40–55 years (38%). Further demographic details of the participants are presented in Table 2.

**Table 2:** Demographic characteristics of participants.

No.	Variables		*N*	%
1	Age groups (year)	18–25	3	12
		25–40	7	27
		40–55	10	38
		55–70	6	23
2	Work experience (year)	3–10	5	19
		10–20	8	31
		20–30	5	19
		30–40	8	31
3	Gender	Male	23	88
		Female	3	12
4	Marital status	Single	6	23
		Married	20	77
5	Educational level	Illiterate	4	15
		Middle school degree	8	31
		Diploma	1	4
		Bachelor	6	23
		Master	4	15
		PhD	3	12

In this study, a total of 1,765 initial codes were extracted during the initial analysis. After removing duplicates, the number of codes was reduced to 978. Following data review and analysis, the concept of psychological resilience among farmers was explored. Subsequently, factors affecting the psychological resilience of farmers living in drought-affected regions were identified and categorized.

### 3.1 Concept of psychological resilience among farmers living in drought-affected regions

Psychological resilience in this context relates to farmers’ capacity to manage the various impacts of drought. It refers to farmers’ ability to cope, adapt, and recover from stress, adversity, and lack of resources caused by drought.

### 3.2 Factors affecting psychological resilience of farmers living in drought-affected regions

The factors affecting the psychological resilience of farmers living in drought-affected regions were classified into two categories and nine subcategories (Table 3). The categories include environmental and intra-individual factors. Environmental factors consist of five subcategories: economic factors, socio-cultural factors, drought adaptation methods, government policies, and infrastructural factors. Intra-individual factors contain four subcategories: personal characteristics, health factors, psychological factors, and perception and knowledge.

**Table 3:** Codes, subcategories, and main categories of factors affecting the psychological resilience of farmers living in drought-affected regions in Iran.

Categories	Subcategories	Selected codes
Environmental factors	1. Economic factors	Having non-agricultural income Insurance coverage
	2. Socio-cultural factors	Emotional support Community bonding
	3. Drought adaptation methods	Water management Planting management Use of new technology
	4. Government policies	Government support Government oversight of legislation
	5. Infrastructural factors	Access to resistant seeds Appropriate transportation Access to agricultural equipment
Intra-individual factors	1. Personal characteristics	Marital status Level of education
	2. Psychological factors	Positive view Sense of belonging Self efficacy
	3. Health factors	Physical health Mental health
	4. Perception and knowledge	Awareness of the consequences of drought Awareness of weather patterns

#### 3.2.1 Environmental factors

In this research, participants discussed factors impacting psychological resilience directly and indirectly via environmental influences. It is essential to comprehend these factors to enhance farmers’ resilience, potentially influencing their vulnerability to drought consequences. The next part expands on five subcategories of environmental factors.

##### 3.2.1.1 Economic factors

Many participants highlighted economic factors such as agricultural and non-agricultural income, land ownership and size, agricultural expenses, and medical and insurance costs as critical influences on their psychological resilience. When farmers have financial security, they can face the future with confidence and experience calmness. They expressed that feeling financially insecure significantly increased their susceptibility to despair. Additionally, interviewees noted that inadequate product insurance coverage, inconsistent reimbursement from insurance firms for losses, absence of medical insurance coverage, and lack of workplace accident insurance made farmers feel less confident about their future.

“*Many of the trees on my farm have dried up, and I have not had a harvest even close to what I had a quarter of a year ago. Moreover, the current prices are insufficient to offset the land expenses. It is now challenging to sustain my livelihood, and I feel embarrassed in front of my family. At times, it even crosses my mind to seek alternative employment…*” (Participant 12).

##### 3.2.1.2 Socio-cultural factors

Based on the findings, emotional support from family and friends, characterized by understanding, empathy, and attentive listening during difficult periods, was highlighted. Furthermore, engagement in local community groups was noted to maintain cultural connections, traditions, and a sense of identity. Participants noted that residing in a village could offer advantages such as access to green spaces, spacious homes, consumption of organic foods, and isolation from urban noise and pollution, all of which could enhance the psychological resilience of farmers. Moreover, the strong work ethic and resilience demonstrated by rural farmers were cited as significant contributors to their psychological resilience. Farmers also emphasized that involvement in traditional ceremonies and festivities could instill a sense of purpose, significance, and shared values, all crucial elements in bolstering their psychological resilience.

“… *My family, particularly my wife, truly comprehends me. Even with household responsibilities, she diligently assists me with animal husbandry and* var*ious agricultural tasks*…” (P 14).

##### 3.2.1.3 Drought adaptation methods

Based on the participants’ statements, utilizing new agricultural technology and equipment, along with effective management of planting and dealing with water, soil, and pests as well as institutional supports can alleviate psychological stress and anxiety stemming from water scarcity and agricultural issues exacerbated by drought. Through the implementation of drought adaptation strategies, farmers may feel more empowered and capable of addressing drought impacts. Furthermore, the sense of control and readiness fostered by these adaptation measures is crucial in enhancing psychological resilience.

“… *Employing efficient agricultural management strategies can instill feelings of control, stability, and hope among farmers*…” (P 25).

##### 3.2.1.4 Government policies

Another significant category identified in this study is government policies. Participants stated that aligning governmental policies with drought crisis management can alleviate the stress and anxiety experienced by farmers. Moreover, by overseeing the execution of government policies and initiatives, customized services can be accessed according to the specific requirements and severity of drought challenges faced by farmers, thereby contributing to their psychological resilience.

“… *the lack of supervision on unauthorized water well drilling, unexpected price fluctuations, and inequitable distribution of credit among farmers are issues that have left us feeling frustrated, disheartened, and angry*…” (P 18).

##### 3.2.1.5 Infrastructure factors

Certain participants noted that having access to dependable infrastructure and essential services like transportation networks, communication systems, healthcare facilities, and public services enhances farmers’ capacity to manage the diverse impacts of drought. Infrastructure can instill a feeling of security, and stability, and consequently play a significant role in bolstering psychological resilience. Moreover, infrastructure that facilitates access to agricultural resources and services helps in alleviating stress and anxiety during drought periods, leading to an enhancement in the general welfare of farmers and rural communities.

*“… having secure access to resources and infrastructure puts me in a stronger position to confront challenges. It could improve my resilience against market shifts and environmental variations. I cannot engage in battle empty-handed…”* (P 11).

#### 3.2.2 Intra-individual factors

The second category identified in this research is intra-individual factors. Intra-individual factors pertain to the internal traits of individuals that influence their psychological resilience. Participants noted that these factors play a role in a farmer’s resilience by contributing to their adaptation to challenges, stress management, and sustaining a positive mindset amidst drought impacts. Additionally, they enhance farmers’ capability to cope with and navigate challenging circumstances and maintain their mental health. This category encompasses four subcategories, which are explained below.

##### 3.2.2.1 Personal characteristics

The research findings indicated that certain elderly farmers have gathered years of experience and wisdom, promoting their capacity to confront challenges and adjust to drought circumstances. Conversely, some older farmers viewed their physical constraints and health issues as obstacles to their resilience. Married farmers highlighted that emotional support, collaborative decision-making, and task delegation positively impacted their psychological resilience. Higher education was noted to enhance problem-solving capabilities, foster creative thinking, and improve decision-making skills. Furthermore, farming experience was highlighted as a factor that bolsters feelings of trust and competence.

“… *In the past, we faced prolonged drought periods, but thankfully, we overcame them. I have experienced drought since childhood and learned how to handle it. Naturally, if my health permits and I can still work*…” (P 9).

##### 3.2.2.2 Health factors

Participants emphasized the impact of health in physical and mental on their psychological resilience. Farmers with good physical and mental health exhibited higher energy levels, a robust immune system, enhanced stress management skills, and a positive outlook, all of which improved their psychological resilience in confronting drought challenges. Conversely, illnesses or chronic mental conditions could lead to increased stress and pressure, reducing the psychological resilience of the farmers.

“… *I rely on drugs to alleviate the joint pains resulting from my work. However, this has resulted in additional physical and mental issues,* reducing *decision-making abilities and straining relationships*…” (P 8).

##### 3.2.2.3 Psychological factors

Most interviewees expressed that self-efficacy, job satisfaction, creativity and innovation, and a sense of belonging to a particular place play a significant role in their psychological resilience. Participants highlighted that having faith in their capability to surmount obstacles and reach objectives, possessing self-assurance, motivation, and determination, actively seeking solutions, and managing stress and anxiety are crucial elements that shape the psychological resilience of farmers during drought periods. These factors can foster a positive perspective and assist individuals in maintaining a hopeful mindset in challenging circumstances.

“… *I have a deep attachment to residing in the village, where my ancestors resided. My identity is inseparable from this place; my roots run deep here. The entire village community stands by me. Together, we can overcome any challenge with mutual support*…” (P 4).

##### 3.2.2.4 Perception and knowledge

Another subcategory of intra-individual factors is perception and knowledge. As indicated by the research findings, comprehending the nature of issues and being aware of the resources and alternatives available enhances problem-solving abilities. Understanding the consequences of drought, being knowledgeable about factors influencing resilience, and being informed about new agricultural techniques and equipment enable farmers to adjust their approaches in reacting to outcomes. Participants believe that having adequate knowledge and awareness of the ramifications ultimately helps farmers overcome obstacles and strengthen their psychological resilience.

“… *thanks to news sources and the Internet, I have gained insight into the severe impacts of drought on agriculture. Without this knowledge, it might have been too late for preventive measures. However, now I feel more motivated and my mind is at ease*…” (P 17).

## 4 Discussion

This study represents one of the initial investigations to explore the psychological resilience of farmers during drought conditions. Through qualitative research, we identified the concepts and factors influencing the psychological resilience of farmers living in drought-affected regions of Iran were identified. The study results led to the classification of these factors into two main categories and nine subcategories. The categories encompass environmental factors and intra-individual factors. Environmental factors are divided into five subcategories including economic factors, socio-cultural factors, drought adaptation methods, government policies, and infrastructural factors. Intra-individual factors consist of four subcategories, including personal characteristics, health factors, psychological factors, and perception and knowledge.

### 4.1 Economic factors

Participants recognized the impact of economic factors on their psychological resilience. This influence is well-documented in various studies. For instance, a systematic review highlighted the significant relationship between financial assistance provided to farmers affected by drought in tropical Asia and their resilience (Goodwin et al., 2022). A 2020 study revealed that financial stress diminishes life satisfaction among farmers (Heo et al., 2020). Other research indicated that land ownership affects farmers’ resilience (Mehra et al., 2022). Economic factors have been underscored as a crucial resource that bolsters resilience (Wong et al., 2023). In a study examining psychological resilience among Chinese residents during the COVID-19 epidemic, financial status and economic factors were influential elements impacting their psychological resilience (Shen et al., 2022). In summary, significantly impact farmers’ psychological resilience by strengthening their motivation.

### 4.2 Socio-cultural factors

The study highlighted the significant impact of socio-cultural factors on farmers’ psychological resilience. Similarly, researches underscore the pivotal role of socio-cultural factors in influencing individuals’ ability to adapt and progress amidst challenging circumstances (Gao et al., 2023; Darnhofer et al., 2010). Enhancing social support is advantageous for the mental well-being and resilience of male farmers (McLaren and Challis, 2009). During the COVID-19 pandemic in Japan, social networks were identified as critical attributes contributing to farmers’ resilience (Yoshida and Yagi, 2021). The significance of resources and social cohesion in fostering resilience among farmers and their families has been highlighted (Caldwell and Boyd, 2009). Socio-cultural factors are thus critical for farmers’ psychological resilience.

### 4.3 Drought adaptation methods

Consistent with our findings, a study reveals that farmers exhibit increased psychological resilience through mitigation strategies like mulching, new irrigation techniques, and other innovations (Kgopa, 2022). Furthermore, in another study examining the impact of drought on rural families, it is noted that families use coping mechanisms to enhance psychological resilience, suggesting the need for their development (Caldwell and Boyd, 2009). Another study recommend implementing strategies such as altering agricultural techniques and practices, diversifying livelihoods, and seeking new resources to enhance farmers’ resilience to climate-related risks (Panchi Robles, 2019). Overall, drought adaptation strategies are linked to improved psychological resilience among farmers.

### 4.4 Government policies

Government policies emerged as significant in influencing psychological resilience. In previous studies, this factor has been highlighted as influencing psychological resilience. For instance, a study indicated that government interventions and support play a significant role in enhancing the resilience of rice farmers in India (Duncan et al., 2017). Additionally, another study underlined that government initiatives and policies are crucial in bolstering the psychological resilience of farmers (Xie et al., 2023). Government policies and programs can enhance the mental health of farmers (Jones-Bitton et al., 2020). Therefore, government policies are critical factors influencing farmers’ psychological resilience.

### 4.5 Infrastructural factors

Validating our findings, studies highlight that the absence of infrastructure and technologies reduces farmers’ resilience to climate change and drought conditions (Javadinejad et al., 2021). Infrastructural factors are recognized as significant contributors to resilience among rural Australians (Buikstra et al., 2010). In Bejestan, infrastructural factors were identified as crucial components of residents’ resilience against drought (Farahani and Jahansoozi, 2022). Thus, the Lack of Infrastructure and Technology diminishes Farmers’ Psychological Resilience, aligning with findings from related studies.

### 4.6 Intra-individual factors

In addition to environmental factors, another finding of this study is the significance of intra-individual factors. Similar to research on understanding resilience in stressful work environments, this study underscores the pivotal role of intra-individual factors in psychological resilience (Rees and Breen, 2015).

### 4.7 Personal characteristics

Personal characteristics are a subset of intra-individual factors. Research indicates that demographic characteristics such as education level and gender influence resilience (Mohamed Ludin, 2018). For instance, education level and age significantly impact farmers’ resilience (Xie et al., 2023). Another study identified age as an effective factor in psychological resilience (Chen et al., 2020). Additionally, research has shown that age contributes to increased life satisfaction and positive emotions among farmers affected by drought (Forouzani et al., 2024). In summary, intra-individual factors, including personal characteristics like education and age, are key determinants of farmers’ psychological resilience.

### 4.8 Health factors

Health factors, encompassing both physical and mental health, significantly shape psychological resilience. Studies highlight that psychological resilience depends on mental health and overall well-being (Greenhill et al., 2009). In Similar research, it is noted that higher levels of stress, anxiety, and depression correlate with lower resilience, emphasizing the link between farmers’ general health and their psychological resilience (Jones-Bitton et al., 2020). Furthermore, researchers in a study indicated that health status is a stabilizing factor amidst stressors, underscoring the importance of good mental health for resilience (Bondy and Cole, 2020). Consequently, health factors play a crucial role in shaping farmers’ psychological resilience.

### 4.9 Psychological factors

Psychological factors are the other findings of the study. The impact of these factors has been confirmed in numerous studies. For instance, in a similar study, factors influencing farmers’ psychological resilience were identified as a passion for agriculture, hope, courage, acceptance or tolerance, and a coherent belief system (Kgopa, 2022). Additionally, another study confirmed that farmer families try to enhance their resilience by emphasizing optimism, positive appraisal, and avoiding negative influences (Caldwell and Boyd, 2009). In a research study, cognitive factors such as self-efficacy and a sense of belonging to a place were noted to influence the resilience of farmer families in drought conditions (Savari and Moradi, 2020). In summary, psychological factors are the most critical factor for the farmer’s psychological resilience, aligning with findings from prior research.

### 4.10 Perception and knowledge

Perception and knowledge also impact psychological resilience. Research indicates that farmers’ limited understanding of climate change affects their resilience (Kgopa, 2022). Other researchers have highlighted the significance of understanding El Niño-Southern Oscillation (ENSO) forecasts in determining farmers’ resilience against drought linked to this phenomenon (Keil et al., 2008). Therefore, perception and knowledge are crucial for farmers’ psychological resilience.

Recent studies emphasize the dynamic and adaptable nature of psychological resilience, moving away from viewing it solely as an individual trait (Denckla et al., 2020). This study’s findings have the potential to enhance farmers’ psychological resilience in addressing drought disasters, fostering adaptability, reducing migration, and improving quality of life for farmers.

### 4.11 Strengths and limitations

One of the key strengths of this study is its qualitative research approach, which allowed for a deeper exploration and enhanced comprehension of the psychological resilience of farmers living in drought-affected regions. This approach complemented existing quantitative research, providing a richer and more nuanced understanding of the phenomenon. Another strength lies in the diversity of participants, who brought necessary experience and knowledge about farmers’ psychological resilience in drought-affected regions.

However, a limitation of this research is the inability to generalize the findings to other societies due to the specific context of farmers in Iran. Nevertheless, the insights gained from this study may still be applicable to countries with similar contexts.

## 5 Conclusion

This study identified environmental factors and intra-individual factors affecting the psychological resilience of farmers living in drought-affected regions. The findings can contribute to the identification and analysis of possible scenarios to form and strengthen the psychological resilience of farmers in these regions. Based on the research results, it is recommended to provide the necessary interventions to improve farmers’ mental health levels and prevent mental health disorders resulting from the adverse effects of drought.

Moreover, suitable intervention strategies, such as providing training and education on drought adaptation methods and managing agricultural and healthcare expenses. Further research is needed to conduct longitudinal studies that provide a more comprehensive understanding of the evolving nature of farmers’ psychological resilience. Additionally, it is crucial to evaluate the effectiveness of targeted support programs aimed at enhancing the resilience of farmers in the face of climate change. Furthermore, additional research is required to explore this concept across various disasters and other occupation groups in future studies.

## Data Availability

The raw data supporting the conclusions of this article will be made available by the authors, without undue reservation.
