# Supplementary material for: Exploring factors affecting psychological resilience of farmers living in drought-affected regions in Iran: a qualitative study
Source: Front Psychol. 2024 Aug 27;15:1418361. doi: 10.3389/fpsyg.2024.1418361 (PMC11403586; doi:10.3389/fpsyg.2024.1418361)
Supplement: Supplementary file 2 [file Data_Sheet_2.docx]

**Interview guide**

**Research title: " Exploring Factors Affecting Psychological Resilience of Farmers Living in Drought-Affected Regions in Iran: A Qualitative Study"**

The name of the interviewer: Azadeh Tahernejad, PhD student of health in disasters and emergencies, School of Public Health and Safety, SBMU, Tehran, Iran.

**The purpose of the research**

this research aims to explore factors affecting the psychological resilience of farmers living in drought-affected regions of Iran".

**The nature of the interview**

The interview is conducted to receive and use the experiences of experts and farmers regarding the Factors Affecting the Psychological Resilience of Farmers Living in Drought-Affected Regions in Iran. There are no right or wrong answers in this interview, but the researcher only seeks to know the perceptions and experiences of mask users in this field. It is expected that the interview will last about 25-45 minutes. If the interview is not completed in the mentioned time, the interviewee is requested to add some time to the interview if there is enough time.

**Keeping confidential documents**

All the interviewees are assured that the confidentiality of the answers will be fully respected the data will be used only in line with the goals of the research and only the researcher will have access to the information. In any case, under no circumstances the interviewee's name won’t be included anywhere.

For more in-depth investigation and analysis, with the knowledge and consent of the interviewee, a voice recorder will be used to record the voice. It is worth mentioning that the data extracted from interviews are considered completely confidential and can only be identified by a code or number.

**voice recording**

Is it possible for me to get help from recording this interview?

Do you have any questions before the interview?

- Turn on the recorder after the participant's agreement

**1- Your professional experience**

I would like to know your demographic information and your background before discussing the main subjects under investigation.

- Age: ……...
- Gender: ……...
- Marital status: ……...
- Education level: ……...
- Work experience: ……...

**2- The main topics and questions of the interview**

- For farmers:

- Can you describe your experience living and farming in a drought-affected region?

- What are the main challenges and stressors you face as a farmer in this environment?

- How do you cope with the difficulties and maintain your well-being during times of drought?

- What personal, social, or community resources help you manage the impacts of drought?

- Can you share examples of how you have been able to adapt and remain resilient in the face of drought?

- What support or interventions do you think would be most helpful for farmers like yourself in drought-affected areas?

- For experts:

- From your experience working with farmers in drought-affected regions, what are the key factors that influence their psychological resilience?

- What are the major challenges and stressors that farmers in these regions commonly face?

- What coping strategies and resources have you observed farmers using to maintain their well-being?

- In your opinion, what individual, social, and community-level factors contribute to stronger psychological resilience among farmers?

- What types of support or interventions do you think would be most effective in enhancing the resilience of farmers in drought-affected areas?

- How can policymakers and service providers better address the mental health and psychosocial needs of farmers living in drought-prone regions?

To probe and deeply investigate people's experiences to reach saturation, the researcher uses how and why. Who or what questions? Furthermore, questions are formed during the interview to maintain the dynamics of the interview and guide it toward the interview goals.
